# Prevalence of Single and Multiple Leading Causes of Death by Race/Ethnicity Among US Adults Aged 60 to 79 Years

**DOI:** 10.5888/pcd14.160241

**Published:** 2017-10-19

**Authors:** James Davis, Janell Penha, Omar Mbowe, Deborah A. Taira

**Affiliations:** 1 Office of Biostatistics and Quantitative Health Sciences, John A. Burns School of Medicine, Honolulu, Hawaiʻi; 2 Department of Pharmacy Practice, Daniel K. Inouye College of Pharmacy at the University of Hawaiʻi at Hilo, Hilo, Hawaiʻi

## Abstract

**Introduction:**

Diabetes, cancer, cardiovascular disease (CVD) (coronary artery disease, heart attack, and angina pectoris), and chronic lung disease (emphysema, chronic bronchitis, and chronic obstructive pulmonary disease) are major causes of death in the United States. The objective of this study was to assess racial/ethnic differences in the prevalence of these conditions as cause of death among people aged 60 to 79 years with one or more of these conditions.

**Methods:**

We used data on the prevalence of major chronic conditions from the National Health Interview Survey on 56,290 adults aged 60 to 79 years who reported having any of the chronic conditions assessed in the National Health Interview Survey for 2006 through 2014. We compared trends with age for 11 single and multiple conditions. Analyses employed multinomial logistic regression models.

**Results:**

Hispanics and non-Hispanic blacks had the greatest prevalence of diabetes, and non-Hispanic whites had the greatest prevalence of cancer and chronic lung disease. The prevalence of multiple chronic diseases in an individual varied less by race/ethnicity. An exception was the prevalence of having both diabetes and CVD, which was higher among Hispanics and non-Hispanic blacks than non-Hispanic whites. Non-Hispanic blacks aged 65 years and 75 years had higher odds of having diabetes and cancer than non-Hispanic whites at the same ages. Hispanics had lower odds of having CVD with cancer or chronic lung disease than non-Hispanic whites. Women had a lower age-specific prevalence than men for most of the 11 single and multiple conditions. Most chronic diseases showed an inverse relationship with education and a higher prevalence in the South than in other regions.

**Conclusion:**

Strong racial/ethnic differences exist in the prevalence of single chronic conditions, but differences are lower for prevalence of multiple conditions. Comparing races/ethnicities, the same disease dyads and triads may occur more often in different orders.

## Introduction

The segment of the US population aged 65 years or older has been growing, especially among ethnically diverse groups. Hispanic populations make up a rapidly growing proportion of the US older (>65 y) population (1,2). With this aging of the population, the prevalence of multiple chronic conditions in an individual has increased to approximately 75% of Americans aged 65 or older (3), and this proportion has been increasing among non-Hispanic blacks, non-Hispanic whites, and Hispanics (4).

Multimorbidity — the presence of more than one disease in a person — affects death rates, health-related quality of life, and use of health care. A study of Medicare beneficiaries found that the mean annual health care cost for a person with chronic conditions was $7,172 for 1 condition, $14,931 for 2, and $32,498 for 3 or more (5). Moreover, approximately 66% of total US health care spending is associated with care for people with multiple chronic conditions (3). The most prevalent disease dyads are hypertension in combination with arthritis, diabetes, cancer, or cardiovascular disease (coronary artery disease, heart attack, and angina pectoris) and arthritis in combination with diabetes or cancer (6). The most common triads are arthritis and hypertension in combination with diabetes, cancer, or cardiovascular disease. In addition, a recent study from Denmark documented a high co-occurrence of mental and somatic disorders, emphasizing the complexity of multimorbidity (7).

The prevalence of multiple chronic conditions varies by race/ethnicity. After adjustment for age, sex, and other factors, non-Hispanic blacks have a higher prevalence of multimorbidity than non-Hispanic whites (6,8–11), and Hispanic populations have a lower prevalence of multimorbidity than non-Hispanic whites (6,10). These findings are consistent with what is known as the Hispanic paradox, which refers to the relatively good health of Hispanic people despite their relatively low socioeconomic status (12,13).

We conducted a comprehensive assessment of how the prevalence and nature of the combinations of the leading causes of death in the United States differ among non-Hispanic blacks, non-Hispanic whites, and Hispanics according to age, sex, geographic region, and education level. The goal of this study was to examine racial/ethnic differences in the prevalence of single and multiple chronic conditions in a national sample of older (60–79 y) Americans.

## Methods

We evaluated prevalence data on 56,290 adults aged 60 to 79 years who responded to the National Health Interview Survey (NHIS) from 2006 through 2014. We compared racial/ethnic differences in the prevalence of single and multiple chronic conditions in an individual by age, sex, geographic region, and education. The study population was participants in NHIS for 2006 through 2014. NHIS is a voluntary, cross-sectional household interview survey conducted annually. Sampling and interviewing are continuous throughout each year. NHIS uses a complex, multistage sample design to survey the health of the civilian, noninstitutionalized US population (14). The survey excludes residents of nursing homes and residential care facilities, populations with a high prevalence of chronic conditions (15). We studied NHIS participants aged 60 to 79 years who self-identified as non-Hispanic black, non-Hispanic white, or Hispanic. This age range encompasses the population with the highest prevalence of many major chronic conditions (16). NHIS collects information on health disorders from one randomly sampled adult per family, although additional information is collected on all adults in the family. Participants in our study were those who reported to NHIS that they had been told by a health care provider that they had the following diseases: diabetes, coronary heart disease, a heart attack, angina pectoris, diabetes, emphysema, chronic bronchitis, chronic obstructive pulmonary disease, or cancer (excluding nonmelanoma skin cancer). These diseases are leading causes of death in the United States (16, 17).

All variables except age were classified into categories for analysis. Races/ethnicities were non-Hispanic black, non-Hispanic white, and Hispanic. Geographic regions were the East, Midwest, West, and South. Education was coded as less than a high school diploma, high school diploma or general equivalency degree, some college (with or without an associate degree), and a college degree or professional school degree (eg, MD, DDS, DVM, JD). Chronic conditions were classified into 4 categories: 1) diabetes; 2) cardiovascular disease (CVD) (coronary artery disease, heart attack, and angina pectoris); 3) chronic lung disease (emphysema, chronic bronchitis, and chronic obstructive pulmonary disease); and 4) cancer, excluding nonmelanoma skin cancer. Participants were asked if a doctor or other health care provider had ever told them that they had one or more of these chronic conditions. The presence of chronic bronchitis was based on whether participants had ever been told by a doctor or other health care provider in the past 12 months that they had chronic bronchitis. The presence of the other chronic conditions was based on whether participants had ever been told by a doctor or other health care provider that they had that condition. Participants were classified by the presence of combinations of the 4 chronic conditions. Race/ethnicity was first categorized as Hispanic/Spanish origin or not (ie, Hispanic), and secondarily by white or black/African American (ie, non-Hispanic white or non-Hispanic black).

All statistical analyses consisted of the strata, cluster, and weight variables provided by the Centers for Disease Control and Prevention to give valid estimates from the complex NHIS design. Preliminary analyses summarized the frequencies of study variables by race/ethnicity. Subsequent analyses used regression models treating the various categories of single and multiple chronic conditions as outcomes. Associations of predictor variables with these outcomes were modeled by using multinomial logistic regression. The models included age, a series of indicators for participating in the survey years (yes or no), indicators for the race/ethnicity categories, and interaction terms between age and the race/ethnicity indicators. Results are presented for the 11 disease categories with the largest samples sizes. Because of the high prevalence of diabetes, and because diabetes is an underlying cause of several chronic conditions, results are stratified by the presence or absence of diabetes. The presence of both cancer and chronic lung disease was the rarest combination, and those results are not included because of small numbers. The probabilities of having the various disease outcomes were calculated from the regression results as described by Agresti (18). Other analyses compared differences in prevalence by race/ethnicity at ages 65 and 75 years. Age was centered by subtracting 65 or 75 from the participants’ ages for these analyses. The results are presented as odds ratios (OR) with 95% confidence intervals (CIs). The multinomial models were extended by adjusting for sex and indicators for the geographic regions, education, and survey year. All analyses were performed with SAS version 9.4 (SAS Institute, Inc), and *P* values at or below .05 were deemed significant.

## Results

Racial/ethnic differences in the prevalence of single and multiple chronic conditions in a person varied by age, sex, region, and education. Most (73.4%) of the 56,290 adults studied were non-Hispanic white. Racial/ethnic differences by age group and sex were within a few percentage points, but differences in prevalence by education levels differed substantially (Table 1). Almost half (48.1%) of the Hispanic population had less than a high school education, and only 11.7% had a college degree or professional school degree. By contrast, 87.2% of non-Hispanic whites and 71.2% of non-Hispanic blacks had a high school diploma. Non-Hispanic whites were twice as likely as non-Hispanic blacks to have a college degree or professional license (29.4% vs 15.6%) and almost twice as likely to have less than a high school diploma (12.8% vs 28.7%). Racial/ethnic differences in the prevalence of chronic diseases were greatest for diabetes and cancer. Thirty percent of non-Hispanic blacks and 29% of Hispanics reported having diabetes compared with 17% of non-Hispanic whites. Cancer prevalence was 8.8% for Hispanics, 12.2% for non-Hispanic blacks, and 16.9% non-Hispanic whites.

**Table 1:** Characteristics of Study Participants (N = 56,290), Leading Causes of Death by Race/Ethnicity, National Health Interview Survey, 2006–2014^a^

Characteristic	Non-Hispanic White	Non-Hispanic Black	Hispanic
**Overall, N (%)**	41,297 (73.4%)	N = 8,513 (15.1%)	N = 6,480 (11.5%)
**Age, y**			
60–69	63.1 (0.3)	65.6 (0.7)	65.8 (0.8)
70–79	36.9 (0.3)	34.4 (0.7)	34.2 (0.8)
**Sex**			
Female	52.7 (0.3)	57.4 (0.7)	54.4 (0.8)
Male	47.3 (0.3)	42.6 (0.7)	45.6 (0.8)
**Education**			
<High school diploma	12.8 (0.3)	28.7 (0.7)	48.1 (0.9)
High school diploma or general equivalency degree	30.9 (0.3)	30.4 (0.7)	23.2 (0.7)
Some college	26.9 (0.3)	25.2 (0.6)	17.1 (0.6)
College or professional school degree^b^	29.4 (0.4)	15.6 (0.5)	11.7 (0.5)
**Chronic disease**			
Cardiovascular disease	16.3 (0.2)	15.7 (0.5)	14.1 (0.5)
Lung^c^ disease	10.8 (0.2)	9.0 (0.4)	6.8 (0.4)
Diabetes	16.9 (0.2)	30.1 (0.6)	29.0 (0.8)
Cancer	16.9 (0.2)	12.2 (0.4)	8.8 (0.4)

a Values are % (standard error) unless otherwise indicated. The percentages for age group, sex, education, and the 4 chronic diseases all differed significantly by race/ethnicity (*P* < .001).

b Professional school degree indicates degrees such as MD, DDS, DVM, or JD as defined in the National Health Interview Survey.

c Lung disease was defined as emphysema, chronic bronchitis, and chronic obstructive pulmonary disease.

**Figure 1:**
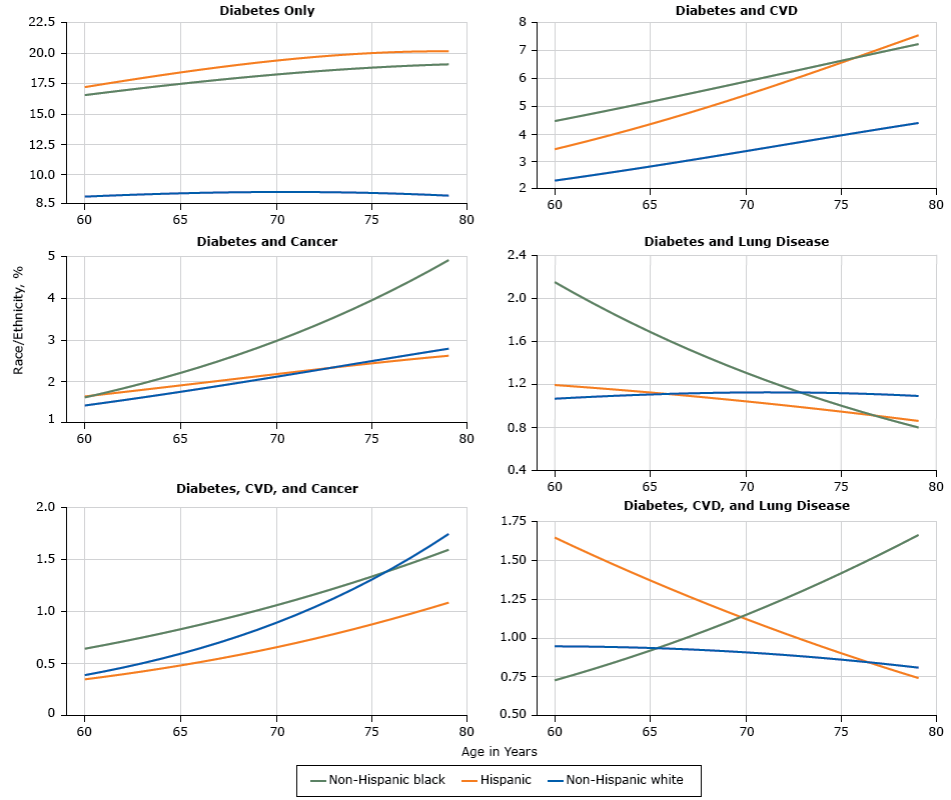
Prevalence of single and multiple chronic conditions that include diabetes among US adults aged 60 to 79 years, National Health Interview Survey, 2006–2014. Abbreviations: CVD, cardiovascular disease; lung, chronic lung disease.

Among adults with diabetes aged 60 to 79 years, the prevalence of diabetes alone or diabetes with CVD was twice as high among Hispanics and non-Hispanic blacks as among non-Hispanic whites (Figure 1). The co-occurrence of diabetes and cancer was similar among non-Hispanic whites, non-Hispanic blacks, and Hispanics at age 60 but was more than twice as high among non-Hispanic blacks by age 79. Co-occurrence of diabetes and lung disease was relatively uncommon. Co-occurrence of diabetes, CVD, and cancer increased with age among the 3 racial/ethnic groups.

**Figure 2:**
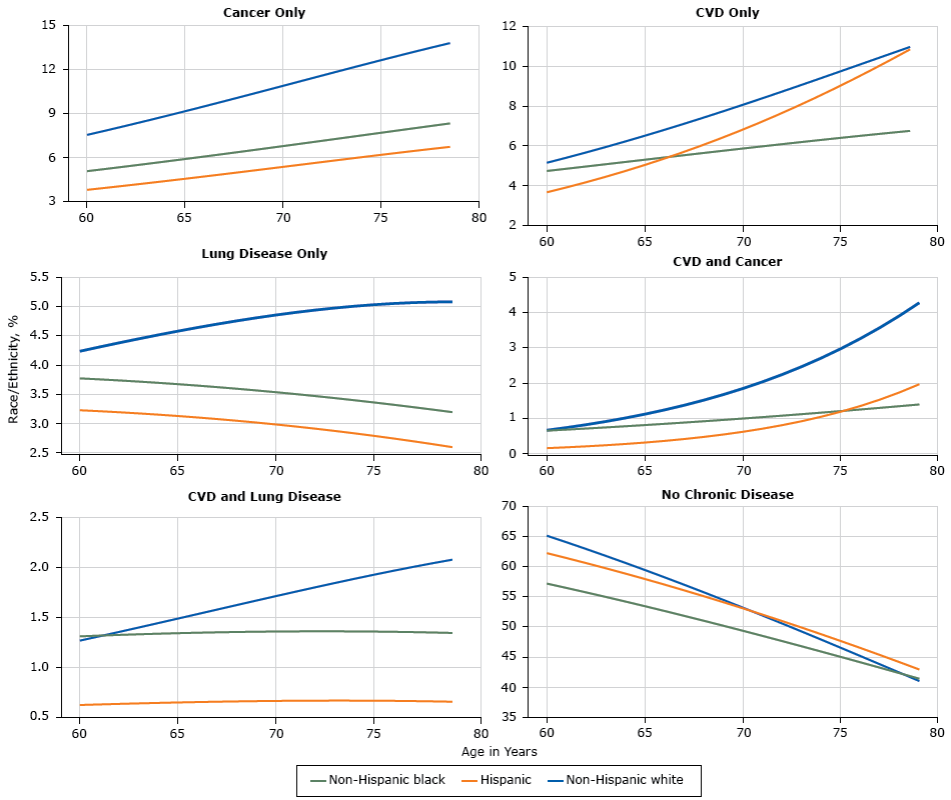
Prevalence of single and multiple chronic conditions that exclude diabetes, National Health Interview Survey, 2006–2014. Abbreviations: CVD, cardiovascular disease; lung, chronic lung disease.

Among non-Hispanic whites the prevalence of multimorbidities that include diabetes was low; however, non-Hispanic whites had a high prevalence of multimorbidities that exclude diabetes (Figure 2). Non-Hispanic whites had the highest prevalence of cancer only or lung disease only. Among Non-Hispanic blacks and Hispanics, prevalence of these 2 conditions singly was lower than among non-Hispanic whites as was prevalence of co-occurrence of CVD and lung disease. At most ages, non-Hispanic whites had the highest age-specific prevalence of CVD only or CVD in combination with either lung disease or cancer. Hispanics had a very low prevalence of CVD in conjunction with cancer. Except for having CVD as a single condition, non-Hispanic whites who did not have diabetes had the greatest increases in prevalence with age.

The prevalence of the 4 chronic diseases we studied varied across the survey years. Prevalence of diabetes increased from 18.0% in 2006 to 19.5% in 2014; lung disease prevalence increased from 9.8% to 12.2%, cancer prevalence increased from 14.7% to 15.7%, and CVD prevalence decreased from 17.5% to 14.2%.

Odds ratios comparing the prevalence of chronic diseases among adults aged 65 and 75 years highlight racial/ethnic differences (Table 2). Most notable were the high odds of non-Hispanic blacks and Hispanics compared with non-Hispanic whites for having only diabetes or diabetes in conjunction with CVD. Non-Hispanic blacks had significantly higher odds than non-Hispanic whites at one or both ages for chronic conditions that include diabetes. The odds ratios for chronic conditions that exclude diabetes presented a different pattern: odds ratios for non-Hispanic blacks and Hispanics were consistently lower than for non-Hispanic whites. Among Hispanics, 9 of 10 comparisons at ages 65 and 75 years were significantly lower. The odds ratios of non-Hispanic blacks for any of the diseases or disease combinations studied decreased from age 65 to 75 years except for the combination of diabetes, CVD, and chronic lung disease, which increased from OR 1.1; 95% CI, 0.8%–1.5% to OR 1.7; 95% CI, 1.1%–2.6%.

**Table 2:** Comparison of Prevalence of Chronic Diseases Among Study Participants Aged 65 Years and 75 Years, National Health Interview Survey, 2006–2014^a^

Chronic Disease	Non-Hispanic Black		Hispanic		Non-Hispanic White	
	65 y	75 y	65 y	75 y	65 y	75 y
Diabetes	2.3 (2.1–2.5)	2.3 (2.0–2.6)	2.2 (2.0–2.5)	2.3 (2.0–2.7)	Reference^c^	
Diabetes and CVD	2.0 (1.7–2.5)	1.7 (1.4–2.1)	1.6 (1.3–1.9)	1.6 (1.3–2.0)		
Diabetes and cancer	1.4 (1.1–1.8)	1.6 (1.2–2.1)	1.1 (0.8–1.5)	1.0 (0.7–1.4)		
Diabetes and lung disease^b^	1.7 (1.3–2.3)	0.9 (0.6–1.5)	1.0 (0.8–1.4)	0.8 (0.5–1.3)		
Diabetes, CVD, and cancer	1.6 (1.1–2.3)	1.1 (0.7–1.5)	0.8 (0.5–1.3)	0.7 (0.4–1.1)		
Diabetes, CVD, and lung disease^b^	1.1 (0.8–1.5)	1.7 (1.1–2.6)	1.5 (1.1–2.1)	1.0 (0.6–1.8)		
Cancer only	0.7 (0.6–0.8)	0.6 (0.5–0.8)	0.5 (0.4–0.6)	0.5 (0.4–0.6)		
CVD only	0.9 (0.8–1.1)	0.7 (0.6–0.8)	0.8 (0.7–0.9)	0.9 (0.7–1.1)		
Lung disease^b^ only	0.9 (0.8–1.1)	0.7 (0.5–0.9)	0.7 (0.5–0.9)	0.5 (0.4–0.7)		
CVD and cancer	0.8 (0.6–1.2)	0.4 (0.3–0.6)	0.3 (0.2–0.5)	0.4 (0.2–0.6)		
CVD and lung^b^	1.0 (0.8–1.3)	0.7 (0.5–1.0)	0.5 (0.3–0.7)	0.3 (0.2–0.6)		

Abbreviation: CVD, cardiovascular disease.

a Values are odds ratio (95% confidence interval).

b Lung disease was defined as emphysema, chronic bronchitis, and chronic obstructive pulmonary disease.

c Results are from a multinomial logistic regression model with “none of the conditions” selected as the comparison group.

In adjusted statistical models, sex, region, and education had strong associations with chronic disorders (Table 3). For most disorders, women had lower odds of disease than men. Compared with the South, other geographic regions had lower odds for many of the chronic diseases. The West was significantly lower than the South in 9 of 11 comparisons of diseases or disease combinations. The West was lower than the Northwest on 6 comparisons and lower than the Midwest on 10. Participants with less than a high school diploma had significantly higher odds for 8 of the 11 conditions than participants with a high school diploma but significantly lower odds for having cancer as a single disorder. Participants with a college degree or professional school degree, by contrast, had significantly lower odds than those with a high school diploma in comparisons of 9 of the diseases or disease combinations and significantly higher odds for having only cancer.

**Table 3:** Comparison of Odds of Having Chronic Diseases Among Study Participants (N = 56,290) by Sex, Geographic Region, and Education, National Health Interview Survey, 2006–2014^a^

Chronic Morbidities	Sex^b^	Geographic Region^c^			Education^d^		
	Female	Northwest	West	Midwest	Less Than High School Diploma	Some College	College Degree or Professional Degree^e^
Diabetes	0.8 (0.7–0.9)	0.7 (0.7–0.8)	0.8 (0.7–0.9)	0.9 (0.8–1.0)	1.2 (1.1–1.3)	0.9 (0.8–1.0)	0.6 (0.5–0.6)
Diabetes and CVD	0.4 (0.3–0.4)	0.8 (0.6–0.9)	0.7 (0.6–0.8)	1.0 (0.9–1.2)	1.4 (1.2–1.7)	0.9 (0.8–1.1)	0.5 (0.4–0.6)
Diabetes and cancer	0.8 (0.7–0.9)	0.8 (0.6–1.1)	0.8 (0.7–1.0)	1.1 (0.9–1.3)	0.9 (0.7–1.1)	0.8 (0.7–1.0)	0.7 (0.5–0.8)
Diabetes and lung disease^c^	1.1 (0.9–1.4)	0.7 (0.5–1.0)	0.5 (0.4–0.7)	0.8 (0.7–1.1)	1.9 (1.5–2.4)	0.8 (0.6–1.0)	0.2 (0.1–0.3)
Diabetes, CVD, and cancer	0.4 (0.3–0.5)	0.6 (0.4–0.9)	0.6 (0.4–0.8)	0.8 (0.5–1.1)	1.5 (1.1–2.1)	0.8 (0.6–1.1)	0.6 (0.4–0.8)
Diabetes, CVD, and lung disease^c^	0.7 (0.5–0.8)	0.8 (0.6–1.1)	0.6 (0.4–0.8)	0.9 (0.7–1.2)	2.8 (2.1–3.7)	1.2 (0.9–1.7)	0.5 (0.3–0.7)
Cancer	0.9 (0.8–0.9)	0.9 (0.8–1.0)	0.8 (0.7–0.9)	0.9 (0.8–1.0)	0.8 (0.7–0.9)	1.1 (1.0–1.2)	1.1 (1.0–1.3)
CVD	0.4 (0.3–0.4)	0.8 (0.7–0.9)	0.6 (0.6–0.7)	0.8 (0.7–0.9)	1.3 (1.1–1.5)	0.9 (0.8–1.0)	0.8 (0.7–0.9)
Lung disease^f^	1.2 (1.0–1.3)	0.7 (0.6–0.8)	0.7 (0.6–0.8)	0.8 (0.7–0.9)	1.5 (1.3–1.8)	0.9 (0.8–1.0)	0.4 (0.4–0.5)
CVD and cancer	0.4 (0.3–0.4)	1.0 (0.8–1.3)	0.8 (0.7–1.1)	0.8 (0.7–1.0)	1.2 (0.9–1.6)	1.0 (0.8–1.3)	0.8 (0.6–1.0)
CVD and lung disease^c^	0.5 (0.5–0.6)	0.6 (0.4–0.8)	0.6 (0.5–0.8)	1.0 (0.7-1.2)	1.8 (1.5–2.2)	0.6 (0.5-0.8)	0.2 (0.2–0.3)

Abbreviation: CVD, cardiovascular disease.

a Values are odds ratio (95% confidence interval). Regression models include age, race/ethnicity, indicators for survey year, and interactions between age and race/ethnicity as covariates. Analyses employed a multinomial logistic regression model with “none of the conditions” selected as the comparison group.

b Reference is male.

c Reference is South.

d Reference is high school diploma or general equivalency degree.

e Professional school degree indicates degrees such as MD, DDS, DVM, or JD as defined in the National Health Interview Survey.

f Lung disease was defined as emphysema, chronic bronchitis, and chronic obstructive pulmonary disease.

## Discussion

The study adds a distinct perspective by restricting analyses to diseases that cause the highest number of deaths. The greatest differences occurred among people with single chronic diseases. Compared with non-Hispanic whites, for example, Hispanics and non-Hispanic blacks had double the prevalence of diabetes. Racial/ethnic differences were less distinct for some conditions including multiple diseases. Non-Hispanic whites trended toward a high prevalence for dyads of CVD with cancer or lung disease. Non-Hispanic blacks had a high prevalence of diabetes with CVD or cancer. The prevalence of multiple chronic conditions is complex; it derives from the prevalence of single conditions, incidence rates of other conditions, and mortality. The same multimorbidity can occur through differing pathways. Racial/ethnic differences in single diseases may lead to more diverse patterns or increased differences in multimorbidities. Differences in the prevalence of dyad pairs and of triad triplets of diseases could become more dissimilar.

Other studies of multimorbidity using NHIS may offer the best comparison with our results. Ford et al (19) conducted an NHIS study of arthritis and the same 4 conditions as our study. That study was designed to analyze co-occurring major, lifestyle-related chronic conditions among adults aged 25 years or older. The number of chronic conditions increased with age, was higher in women than in men, and lowest among Hispanics. We excluded arthritis for 2 reasons (1): to give results specific to conditions with high mortality and (2) to increase the sample sizes for combinations of high-risk conditions. The results illustrate the variability in the prevalence of specific multimorbidities that underlie differences in the number of chronic conditions.

Ward and Schiller (6) analyzed 10 conditions among participants in NHIS 2010 aged 18 years or older. Their study reported that 26% of adults had multiple chronic conditions. Multimorbidity increased with age, exhibited a higher prevalence among women than among men, and was higher among non-Hispanic whites and non-Hispanic blacks than among Hispanics (both black and white). The most common disease dyad in Ward et al’s population was arthritis and hypertension; the most frequent disease triad was arthritis, hypertension, and diabetes. These combinations remained the most prevalent among adults aged 65 years or over. In a recent update, Ward et al (20) reported that 85.8% of adults aged 65 years or over had multiple chronic conditions.

In contrast to Ward et al, our results are restricted to a subset of the NHIS population: an older population with chronic conditions that cause the highest number of deaths. We find in agreement with that study that women tended to have the higher prevalence. The prevalence of single and multiple disorders most often increased with age, although in some instances the prevalence remained stable or decreased with age. Our results illustrate that among racial/ethnic subpopulations trends in prevalence exhibit substantial variability in combinations of diseases.

We also observed complexity in the possible health advantage of Hispanic populations. Of the single diseases studied, Hispanics had the lowest overall prevalence of lung disease and cancer, but they had a high prevalence of diabetes. Among people with multiple disorders, Hispanics experienced a higher prevalence of CVD in conjunction with diabetes than non-Hispanic whites. Hispanics and non-Hispanic whites presented similar trends with age for the dyad of diabetes and cancer. The Hispanic health advantage reported for some chronic diseases did not persist throughout the range of subpopulations with similar combinations of chronic diseases.

Our results underscore differences in the prevalence of multiple chronic conditions by geographical region and education. The South had the highest prevalence for most single and multiple chronic conditions. The southern United States exceeds other regions in levels of poverty and other risk factors (20). Variations in disorders by education were extensive: people with a college degree or professional school degree had a lower prevalence of the 11 chronic conditions we studied than people with less education. Participants reporting less than a high school degree had the highest prevalence of the 4 chronic conditions. Smoking and obesity – major drivers of chronic disease – varied geographically and were inversely related to education and other socioeconomic factors (21–24).

In models adjusted for age, race/ethnicity, survey year, and geographic region, women had a significantly higher prevalence than men did of 10 of 11 chronic disorders. Based on Medicare data, Lochner et al (26) observed that women had a higher prevalence of chronic morbidities across all age groups. They noted a high prevalence of chronic disease among non-Hispanic blacks and Hispanic women. Compared with non-Hispanic white women, we found a significantly higher prevalence among Hispanic women in 8 comparisons.

Studies of other populations add insights into the epidemiology of multimorbidity (25–27). Common approaches have been to analyze the number of chronic conditions or to examine specific disease dyads and triads. Studies vary in the selection of chronic conditions. This variation makes comparisons difficult; however, results do offer insights into the health of the populations studied. Recent studies by the Mayo Clinic of adults from Olmstead County, Minnesota, offer guidelines on how to define diseases in ways that others can apply to their local populations (8,9,26).

Our study had limitations. Although analyses of NHIS data have the strength of a nationally representative sample, the self-report of race/ethnicity and other information is a limitation. Errors in reporting single conditions can lead to compounded errors in classifying adults with multiple chronic conditions. The patterns observed should be interpreted recognizing the changing prevalences of the study diseases across the study years. Undiagnosed disease can also lead to errors in patterns of single and multiple chronic diseases. Misclassification may vary by race and ethnicity. One study, for example, reported that Hispanic populations, especially new immigrants, might not know that they have or fail to report diabetes (27). The study analyses are intricate and are not adjusted for multiple comparisons; the repeated analyses of the same subjects will inflate the type 1 error rates. To increase the number of people with multiple conditions, we aggregated cancers together and combined diverse subgroups into the Hispanic population. A limitation of NHIS is the restriction to noninstitutionalized adults, which means that older institutionalized adults with chronic diseases are less likely to be eligible for participation.

Our results support the strategic framework of the US Department of Health and Human Services (HHS) to improve the health and quality of life for people with multiple chronic conditions (29). A specific goal of the framework was to extend the epidemiology of common disease dyads and triads. Our results add to the understanding from 4 leading causes of death but are limited to diseases self-reported by the noninstitutionalized US population. The 4 diseases are a subset of the conditions in the HHS multiple chronic condition framework, and information on all of the framework’s conditions is not available in the NHIS.

Study findings highlight racial/ethnic differences among the leading causes of death but present a more complicated pattern than one of straightforward group disparities. We found greater racial/ethnic differences in prevalence of single chronic disorders than in multiple disorders emphasizing the importance of public health strategies to prevent the first disease. To prevent the complexity of multimorbidity, additional efforts might target people with a single disease. Non-Hispanic black and Hispanic adults had a high prevalence of diabetes, both alone and combined with CVD. Non-Hispanic whites had nearly twice the age-specific prevalence of cancer without other chronic conditions, and non-Hispanic whites had the highest prevalence of chronic lung disease, both as a single condition and combined with CVD. Across the racial/ethnic groups, disease dyads and triads may evolve through different pathways. The existence of distinct racial/ethnic patterns should be considered in the HHS framework to prevent and address multimorbidity.
